# Simulations of Benzene and Hydrogen-Sulfide Gas Detector Based on Single-Walled Carbon Nanotube over Intrinsic 4H-SiC Substrate

**DOI:** 10.3390/mi11050453

**Published:** 2020-04-26

**Authors:** Muhammad Haroon Rashid, Ants Koel, Toomas Rang, Mehadi Hasan Ziko

**Affiliations:** Thomas Johan Seebeck Department of Electronics, Tallinn University of Technology, Ehitajate tee 5, 12616 Tallinn, Estonia

**Keywords:** carbon nanotube, benzene, hydrogen sulfide, sensor, detector, photocurrent, 4H-SiC

## Abstract

Carbon nanotubes (CNTs)-based sensors have gained significant importance due to their tremendous electrical and physical attributes. CNT-based gas sensors have high sensitivity, stability, and fast response time compared to that of solid-state sensors. On exposure to a large variety of organic and inorganic compounds, the conductivity of CNT changes. This change in electrical conductivity is being used as a detection signal to detect different target molecules. Hydrogen-sulfide and benzene are hazardous gases that can cause serious health issues in humans. Therefore, it is mandatory to detect their presence in industrial and household environments. In this article, we simulated CNT-based benzene and hydrogen-sulfide sensor with a nanoscale semiconductor device simulator—Quantumwise Atomistix Toolkit (ATK). The change in the device density of states, electric current, and photocurrent in the presence of target molecules have been calculated. The change in photocurrent in the presence of target molecules has been proposed as a novel detection mechanism to improve the sensor selectivity and accuracy. This change in photocurrent as well as electric current in the presence of target molecules can be used simultaneously as detection signals. Our intension in the future is to physically fabricate this simulated device and use photocurrent as well as electric current as detection mechanisms.

## 1. Introduction

The detection of hazardous biochemicals and gases in industrial and household environments have been a great concern. It is very significant to monitor the emission of such gases for environmental pollution control, industrial process controls, public safety, and health. The demand for the development of miniature and portable gas sensors is increasing day by day. Nanomaterials, especially one-dimensional nanomaterials like carbon nanotubes, nanoribbons and nanowires, are promising candidates to replace the conventional solid-sate sensors due to their exceptional physical and electrical attributes. These materials have great adsorption capabilities and are hence very suitable to adsorb and detect a wide range of gases [1]. Carbon nanotubes (CNTs) were discovered by Iijima in 1991 [2]. Since their discovery, CNT has attracted the attention of researchers due to their exceptional electrical, mechanical, and thermal attributes that make them highly suitable for real-life applications. CNTs are promising candidates for a wide range of applications such as supercapacitors, flexible heaters, medical devices, sensors, nano-electronics, power storage batteries, electricity transmission, automobiles, and light emitting displays [3,4,5,6,7,8].

Furthermore, the transition of electronic cloud distribution in CNT from uniform to asymmetric circular clouds around its cylindrical structure (creating a π-electrons clouds) makes it highly suitable for electrochemical reactions [9]. CNTs are made up of tiny covalently bonded carbon atoms. They may be in a single layer of carbon atoms (single wall CNT/SWCNT) or multiple layers of carbon atoms (multi wall CNT/MWCNT) [2,10]. The most popular techniques to synthesize carbon nanotubes are chemical vapor deposition, arc discharge, and laser ablation [11,12,13,14,15]. Carbon nanotubes show a unique diversity in their electronic behavior. The chirality and diameter of nanotubes dictate the behavior of CNTs. They may show metallic nature or semiconducting nature [16,17]. Semiconducting SWCNTs change their bandgap inversely with their diameter [18]. CNTs are totally made of surface atoms that make them ideal candidates for the detection of chemicals and gases. The most commonly used mechanism in CNT-based sensors to detect foreign particles is the resistive method. In this method, the resistance of CNT changes after the adsorption of gas or chemical molecules. CNT-based field effect transistors also work on the same principle, except that the current through the channel is controlled by the gate voltage [19].

Moreover, in this article, we used the resistive method to detect hydrogen sulfide (H_2_S) and benzene with SWCNT. H_2_S can be found in abundance in natural gas, sulphur hot springs, and volcanic soil. This gas has bad effect on lungs and respiratory system of humans. It can diffuse into human lungs while inhaling. H_2_S can cause serious damage to human nervous system and eyes [20]. Inhaling of this gas causes halitosis (bad breathing) in humans. A very low concentration of about 0.1 to 0.5 ppm of H_2_S can cause halitosis in humans [21]. Thus, this fact makes it very important to detect this gas even at very low concentrations. Usually, the chromatography technique [18] is used to detect and diagnose halitosis in humans. However, this technique is time consuming and expensive. Nanomaterials based sensors are very promising candidates for the replacement of the chromatography technique due to their small size and low cost [22].

Furthermore, benzene is another hazardous gas and the major sources of benzene are petroleum products, automobile exhaust, building materials, and industrial discharges [23]. It can cause nausea, cough, vomiting, and dizziness in humans. Benzene can enter into a human’s body through inhaling [24,25,26]. Therefore, detection of benzene in industrial as well as household environments is mandatory. Conventional solid-state gas sensors are not so accurate to detect a very low concentration of such gases and require high operating temperature [27,28,29,30]. CNTs have emerged as promising candidates to cope with the challenges of selectivity, accuracy, and sensitivity to detect a wide range of organic/inorganic molecules.

In this article, SWCNT over intrinsic 4H-SiC substrate has been used to detect H_2_S and benzene molecules. A novel method (change in photocurrent) has been added along with the existing one (change in electric current) to improve the device accuracy to detect H_2_S and benzene molecules. The work presented in this article is totally computational-based. All the simulations have been done in a nanoscale semiconductor device simulator—Quantumwise Atomistix Toolkit.

## 2. Materials and Methods

In this section, comprehensive details about the used materials and simulated devices have been given. All the simulations have been done in a nanoscale electronic device simulator, Quantumwise Atomistix Toolkit (ATK) (QuantumWise, Copenhagen, Denmark). This software package has an inbuilt graphical user interface called Virtual nanolab (VNL). The complete software package ATK-VNL allows the users to model a large variety of nanoscale electronic devices. We simulated SWCNT and deposited it on intrinsic 4H-SiC substrate to detect H_2_S and benzene molecules.

### 2.1. Carbon Nanotube over Intrinsic 4H-SiC Substrate for the Detection of Hydrogen Sulfide and Benzene Molecules

**Figure 1:**
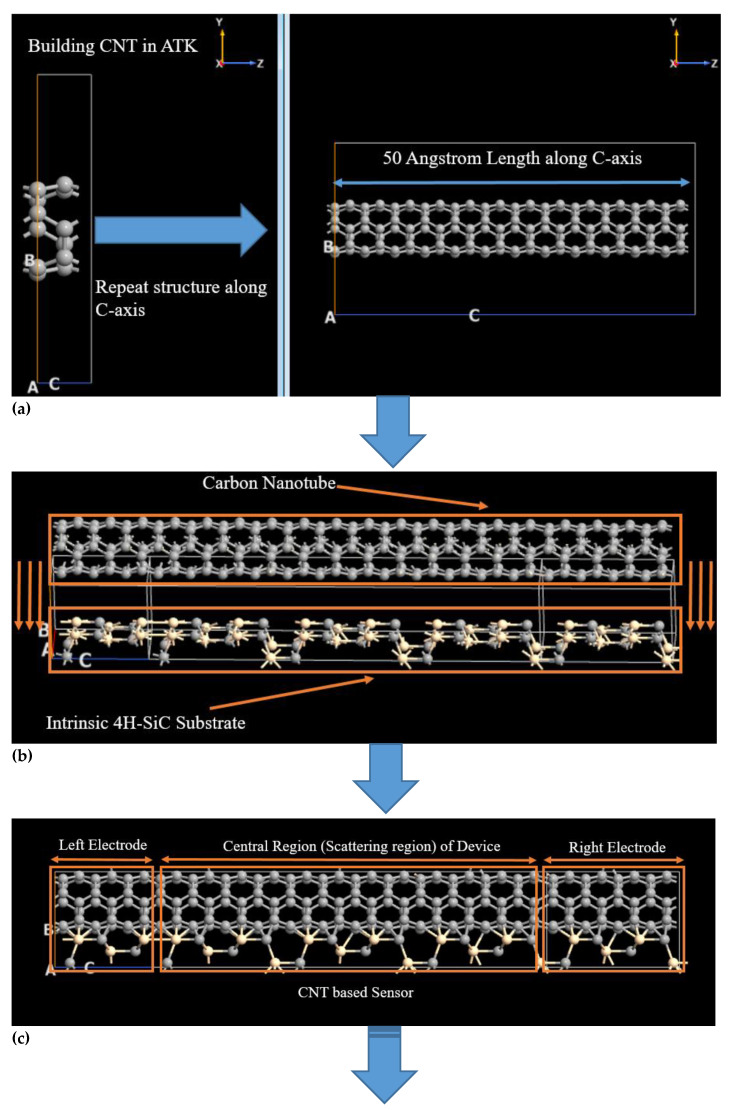

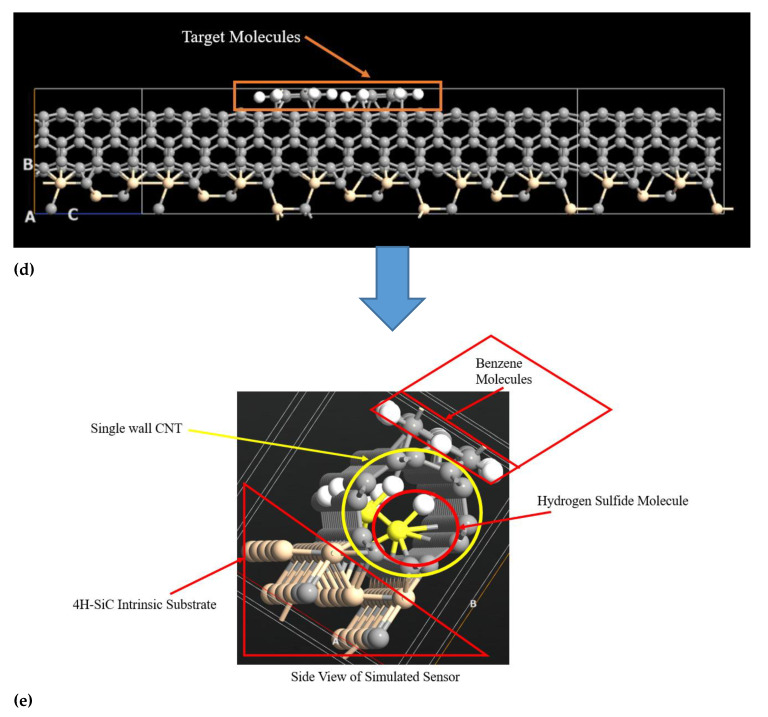
Simulation of single-wall carbon nanotube-based hydrogen-sulfide and benzene detector in Quantumwise Atomistix Toolkit Software.

The constituent materials of the simulated sensor are SWCNT and intrinsic 4H-SiC substrate. SWCNT has been deposited on intrinsic (0001)-oriented 4H-SiC substrate in the ATK-VNL builder tool. In the first step, an inbuilt CNT plugin tool of ATK-VNL software has used to insert the basic structure of CNT, as shown in left image of Figure 1a. Afterward, this basic structure of CNT has been repeated along C-axis to form 50Å long SWCNT, as shown in Figure 1a. In the second step, 4H-SiC has been cut along (0001)-orientation to form a substrate for CNT. After that, CNT has been deposited on this quasi 2-dimensional intrinsic 4H-SiC substrate, as shown in Figure 1b. In the third step, this simulated structure has been converted into device by defining electrodes and central region of the device, as shown in Figure 1c.

Rather than depositing metal electrodes, CNT itself has been used as electrodes to avoid Schottky barrier formation at electrodes and reduce computational time in the simulator. Now, this structure is a CNT-based device that can be used to detect desired target molecules. In the fourth step, the simulated device has been exposed to the target molecules to detect their presence, as shown in Figure 1d. This device has been exposed to two benzene, two hydrogen-sulfide molecules individually, and four H_2_S and two benzene molecules simultaneously in three different experiments. A magnified 3-dimensional side-view of the simulated sensor has been shown in Figure 1e. In this view, SWCNT over intrinsic 4H-SiC substrate has been exposed to hydrogen-sulfide and benzene molecules simultaneously to detect their presence. In physical CNT-based sensors, the target gas molecules can stick to the surface of CNT as well as penetrate into the CNT. CNT having open ends allows the penetration of target molecules into its internal areas. This penetration of target molecule into CNT results in an increase in the sensitivity of the sensor. That is why, the penetration of gas molecules into CNT has also been realized in these simulations, as shown in Figure 1e [31]. The device density of the states (DOS), electric current, and photocurrent have been calculated for four different scenarios, i.e., in the absence of any target, in the presence of only benzene molecules, in the presence of only hydrogen-sulfide molecules, and in the presence of both benzene and hydrogen-sulfide molecules.

### 2.2. Methodology for Simulated Carbon Nanotube Based Benzene and Hydrogen Sulfide Sensor

**Figure 2:**
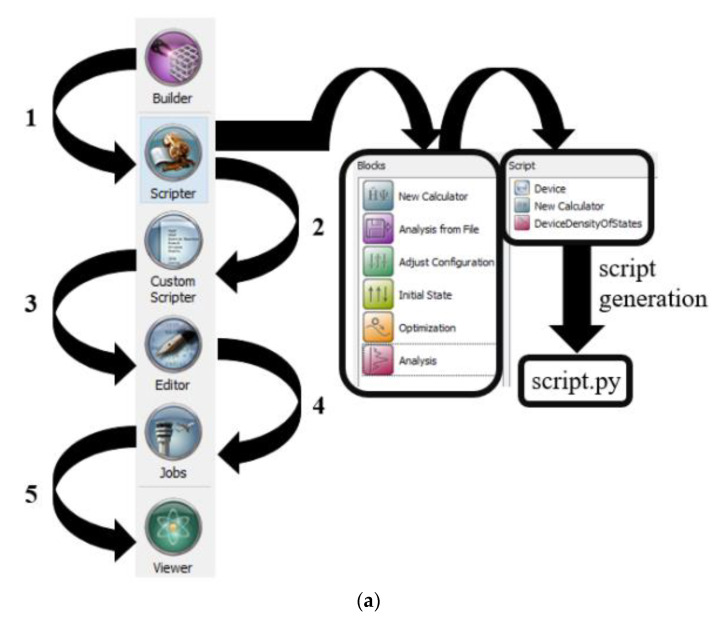

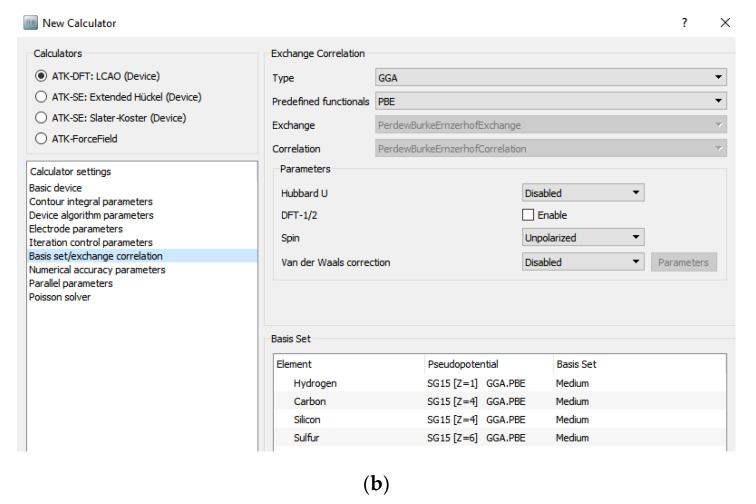
(**a**) Workflow of ATK-VNL software package for the simulation of nanoscale devices; (**b**) Basis set and exchange correlation details for the simulated devices.

All the simulations have been done in a nanoscale semiconductor device simulator, Quantumwise Atomistix Toolkit (ATK). ATK has an inbuilt graphical user interface called Virtual Nanolab (VNL). The ATK-VNL software package allows the users to model the electronic properties of a wide range of organic and inorganic quantum devices [32]. This software is also being used for the simulations of wide range of gas sensors [33,34,35,36,37]. This software has several inbuilt calculators to calculate a wide range of electronic properties of the simulated nanostructures. We used ATK-DFT calculator [38] to compute the electronic properties of our simulated CNT-based benzene and hydrogen-sulfide sensor. ATK-VNL software follows a workflow for the simulations of nanoscale devices. This schematic of this workflow has been shown in Figure 2. In step-1, Builder Tool is used to simulate the desired structure. This tool is a graphical user interface of the software package. In step-2, the simulated structure is transferred to the Scripter Tool. At this stage, an inbuilt calculator and the parameters to be analyzed are added to form a Python code-based script file, as shown in Figure 2. In step-3, the Python code-based script file can be modified and edited by using the Custom Scripter and Editor Tools. In step-4, this Python file is forwarded to the Jobs Tool for execution, as shown in Figure 2. This simulated code can be run at local computing machines or remotely available high-power computing machines. We used remotely available high-power computing (HPC) machines [39] for our simulations. This HPC uses 232 high power computing machines that have 1024 GB of total memory. We used 8-computing nodes to calculate DOS, *I*-*V* curves, and photocurrent for our simulated devices. It took about two weeks to calculate each *I*-*V* curve and photocurrent curve in HPC. The K-point sampling values used in ATK-DFT calculator are *K_a_* = 5, *K_b_* = 5, and *K_c_* = 50 for each calculation. Finally, the simulation results were viewed with the Viewer Tool of ATK-VNL Software. A more comprehensive detail of the workflow used by ATK-VNL can be found at this reference [40]. ATK-DFT calculator has been used for the calculation of DOS, *I*-*V* curves, and photocurrent curves for our simulated devices. Basis Set and Exchange Correlation specifications are shown in Figure 2b.

## 3. Results and Discussions

In this section, the density of states (DOS), *I*-*V* curves, and photocurrent curves of the simulated device have been presented and discussed in detail.

### 3.1. Density of States and Current-Voltage Characteristics Analysis of CNT-Based Device

**Figure 3:**
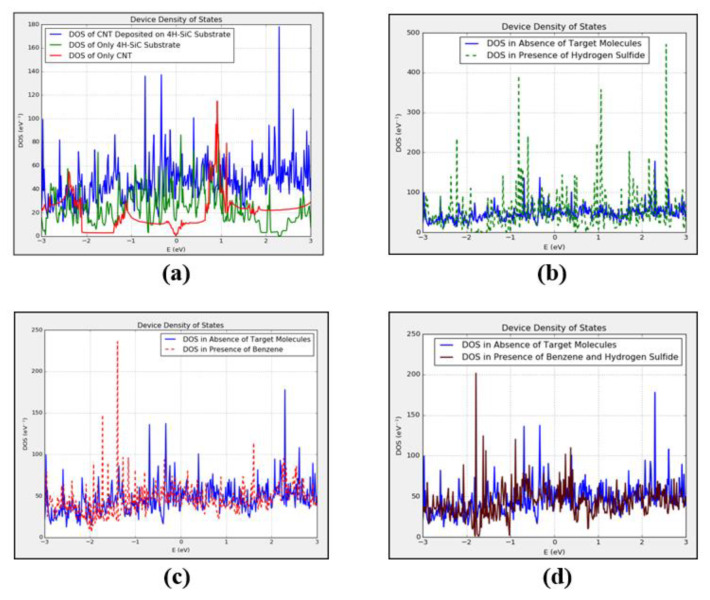
Density of states (DOS) of (**a**) only carbon nanotube-based device; (**b**) device in presence of only hydrogen-sulfide molecules; (**c**) device in presence of only benzene molecules; (**d**) device in presence of both hydrogen-sulfide and benzene molecules.

The DOS of simulated CNT and 4H-SiC substrate have been calculated individually before depositing CNT over 4H-SiC substrate. Then, density of states of only CNT, only 4H-SiC substrate and CNT over 4H-SiC have been compared with each other, as shown in Figure 3a. In this figure, DOS of simulated CNT and intrinsic 4H-SiC have been shown with red and green curves, respectively. Whereas, the DOS of CNT after depositing it over intrinsic 4H-SiC substrate has been shown with a blue curve in Figure 3a. The change in DOS after depositing CNT over 4H-SiC substrate compared to that of only CNT and only 4H-SiC substrate can be observed in Figure 3a.

Moreover, the simulated device shown in Figure 1c has been used as a reference device (in the absence of any target molecules). Later on, this device was exposed to benzene and hydrogen-sulfide target molecules and change in DOS and *I-V* curves were compared with this reference device. In Figure 3b, DOS of CNT-based sensor in the absence of any target molecules has been compared to that of in the presence of two H_2_S molecules. It can be observed in the figure that many new energy states have been added in DOS of the device in the presence of H_2_S gas. Many new sharp energy peaks can be observed in DOS of the device at energy levels of −2.7, −2.3, −2.2, −0.8, 0.9, 1.1, 1.7, and 2.5 eV approximately in the presence of H_2_S gas. These new energy states are not present in the device before its exposure to the target gas, as shown in Figure 3b. Furthermore, a comparison of DOS of the reference device with device in the proximity of two benzene molecules has been shown in Figure 3c. It can be seen in this figure that many new energy states have been added into DOS of the device by the benzene molecules. The new energy states can be viewed at energy levels of −2.3, −2.1, −1.9, −1.7, −1.6, −1.4, −1.2, −1.1, −1, and 1.8 eV approximately. These new energy states will definitely influence the conductivity of the device in the presence of benzene molecules.

Similarly, a comparison of DOS of the reference device with the device in the presence of both hydrogen-sulfide and benzene molecules has been made in Figure 3d. The device has been exposed to two benzene and four H_2_S molecules. It can be observed in this figure that the joint influence of H_2_S and benzene on DOS is very different and unique compared to that of the device in the presence of only H_2_S gas. Many new energy states have been introduced by H_2_S and benzene in the DOS of the device. These new energy peaks can be observed at energy levels of −2.1, −1.8, −1.7, −1.6, and 0.4 eV in the DOS of the device in presence of both H_2_S and benzene molecules in Figure 3d. These energy states were not present in the reference device.

**Figure 4:**
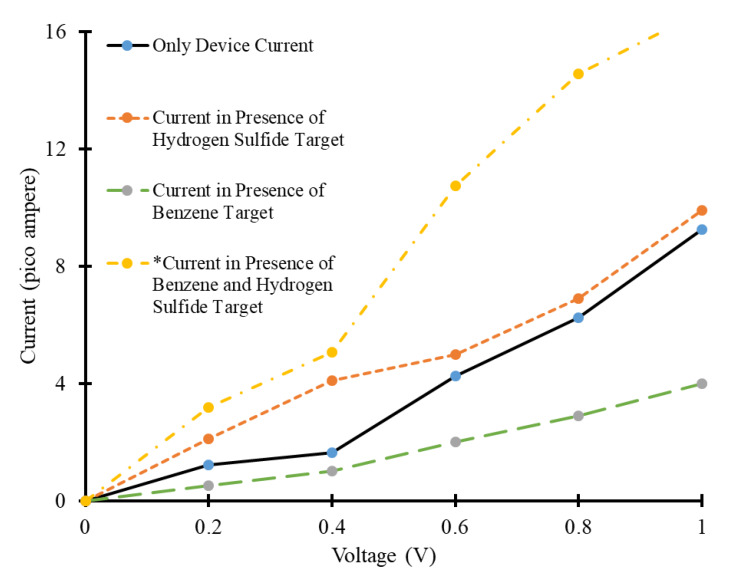
Current-voltage (*I*-*V*) curves of carbon nanotube based simulated device in the proximity of benzene and hydrogen-sulfide as target molecules (*multiply current axis with 10^6^ to get the exact value of current in pico ampere for benzene and hydrogen-sulfide target curve).

The *I-V* characteristics of the simulated device have been calculated for four different scenarios, i.e., in the absence of target molecules, in the presence of only benzene target molecules, in presence of only H_2_S target molecules, in the presence of both benzene and H_2_S target molecules simultaneously. These *I*-*V* curves have been shown in Figure 4. The simulation results revealed that the influence of each target molecule on electric current of the simulated CNT-based device is unique and different. CNTs exhibit a significant change in electric current at the exposure to a wide range of organic/inorganic molecules. The response time of CNT-based sensors is very fast [41,42]. This change in electrical conductivity is used as a detection mechanism to detect foreign adsorbed molecules [43,44].

In this subsection, the change in electric current in the presence of benzene and H_2_S as target molecules has been presented. The simulated device in the absence of any target molecules has been used as a reference device to observe and compare the change in electric current in the presence of target molecules. A voltage bias of 0 to 1 V has been applied at the electrodes of the simulated device and *I*-*V* characteristics have been calculated, as shown in Figure 4. The reference device exhibited a current in the range of 0 to 9.2 pA approximately, as shown with the solid black line in Figure 4. After that, the device was exposed to two H_2_S molecules and change in current has been calculated. It has been observed that exposure of the device to H_2_S gas molecules increased the electric current through the device compared to that of reference of device. The range of current in this case is between 0 to 9.9 pA for the same voltage bias condition, as shown with the orange dotted line in Figure 4. Actually, the adsorbed target molecules act as acceptor or donor for CNT. If they act as donor of charge carriers, they increase the charge carrier’s concentration. Consequently, the conductivity of CNT increases after the adsorption of such molecules. On the other hand, if adsorbed molecules act as acceptors of charge carriers, they reduce the electrical conductivity through CNT [42]. It seems that H_2_S gas molecules act as donors of charge carriers for CNT. Due to which they increase the electric current through CNT after adsorption.

Furthermore, the reference device is exposed to two benzene molecules and change in current has been calculated. The device showed a decrease in electric current at its exposure to benzene molecules compared to that of the reference device, as shown with the green dashed line in Figure 4. The range of the current is between 0 to 4 pA for the same voltage bias condition. Benzene molecules may have acted like acceptors of charge carriers for CNT. Consequently, they reduced the electric current through the CNT, as shown in Figure 4. Whereas, the device exhibited a very high current at its exposure to two benzene and four H_2_S molecules simultaneously. The range of the current in this case is between 0 to 16.678 mA, approximately. The possible reason of this high current could be the presence of more H_2_S gas molecules (which act like donors) compared to that of benzene molecules (which act like acceptor). Consequently, the overall effect of change in charge carrier concentration in CNT is dominated by H_2_S and a large increase in current is observed. The joint influence of both target molecules on electric current is unique and different from the last two cases.

**Figure 5:**
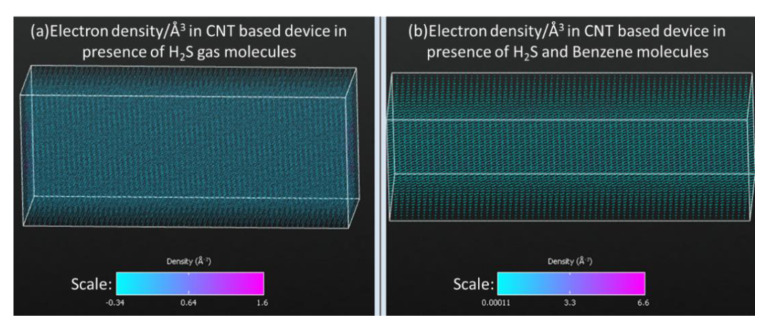
Electron density/Å^3^ of simulated device in presence of (**a**) only H_2_S gas molecules; (**b**) both H_2_S and benzene molecules.

Moreover, electron densities of the simulated device have been calculated to investigate the reason of very large values of electric current through the device at its exposure to both benzene and hydrogen-sulfide molecules simultaneously. At bias voltage of 0.6 V, electron densities have been calculated for all the previously discussed scenarios (in presence of different target molecules). It has been observed that the joint influence of the benzene and hydrogen-sulfide target molecules on the electron density of the device is very strong compared to that of all other cases. At the exposure to benzene and hydrogen-sulfide molecules, high electron density/Å^3^ has been observed, as shown in Figure 5b. In all other scenarios, the values of electron densities/Å^3^ are quite low and approximately in the same range that have been shown in Figure 5a. That is why in all other cases the change in current values are quite low.

Furthermore, in the physical environment the influence of the presence of other gases like hydrogen, oxygen, carbon dioxide, and humidity cannot be neglected. In the simulator, a controlled environment has been used to detect benzene and hydrogen-sulfide gases. In physically fabricated device, a careful calibration is required of to get the actual change in electric current in the presence of only desired gases. For this purpose, the physical device can be first exposed to only desired gases in a controlled environment and change in current is measured. Then, the same device is exposed to the target gases in the physical environment for real-time detection of gases. The difference in electric currents in controlled and uncontrolled environment can be used to get the actual values of current for the detection of desired gases.

### 3.2. Photocurrent Analysis of Carbon Nanotube Based Benzene and Hydrogen Sulfide Detector

In this article, we proposed a novel additional mechanism (change in photocurrent) to be added with the conventional chemical detection mechanism (change in electric current) to improve device selectivity and accuracy for the detection of desired target molecules. The simulated CNT-based sensor has been illuminated by AM1.5 solar spectrum [45] in the presence of benzene and H_2_S targets. The change in photocurrent in the presence of these targets has been measured and compared to that of the device in the absence of target molecules.

However, we could not achieve significant values of photocurrent for our simulated device due to some limitations of the simulator (in controlling polarization of incident light and other related parameters). The generation of photocurrent in CNT is also dependent upon the polarization of incident light [46,47]. CNT has been accepted as an active photo-responsive material. It has several optical attributes like ultraviolet absorption, polarization selectivity, and infrared absorption [48,49,50,51]. It is possible to use this mechanism of change in photocurrent as an additional mechanism along with the conventional techniques to improve the device selectivity and accuracy. In our simulations, we bombard the photons having energy values between 0 to 5 eV on CNT to generate photocurrent. In physical device, a light emitting diode can be used as a light source to illuminate CNT and change in photocurrent as well as electric current in the presence of different target molecules can be measured.

**Figure 6:**
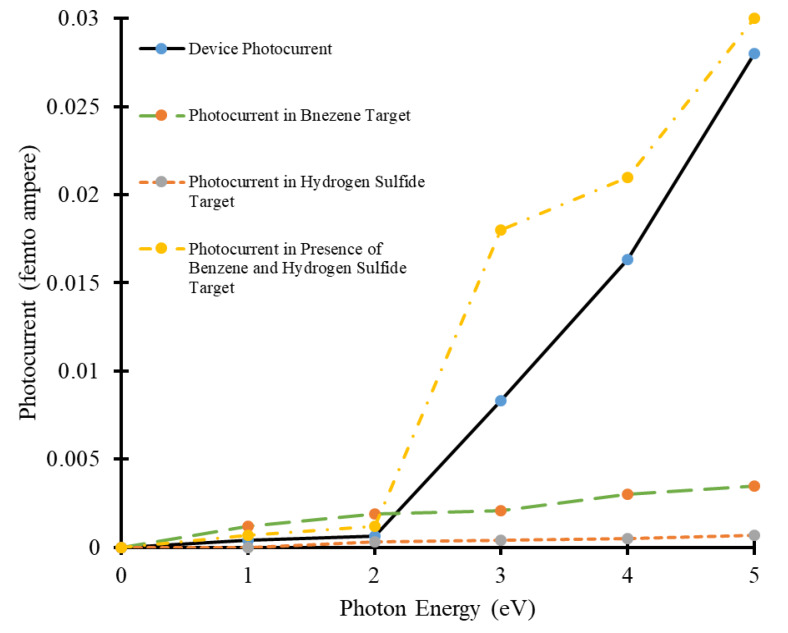
Photon energy vs. photocurrent curves of carbon nanotube-based device in presence of benzene and hydrogen-sulfide target molecules.

Moreover, photocurrent versus photon energy has been shown in Figure 6. It can be observed that the magnitudes of photo-generated current are different for different target molecules. In all cases, an increase in photocurrent with an increase in photon energy has been observed, as shown in Figure 6. The values of photocurrent in the device in absence of target molecules are between 0 to 0.028 fA. Whereas, the range of photo-generated current in the presence of two benzene target molecules is between 0 to 0.0035 fA, as shown with a green dashed line in Figure 6. In the presence of two H_2_S molecules, the range of photo-generated current is between 0 to 0.0007 fA, as shown with the orange dashed line in Figure 6. The highest values of photocurrent was observed when the device was exposed to two benzene and four H_2_S molecules simultaneously. The range of photo-generated current in this case is between 0 to 0.03 fA, as shown with the yellow dotted-dashed line in Figure 6. Although, all these values of photo-generated current are quite low. Still, these values can be extracted and used to detect gas targets in the physical device.

Furthermore, the purpose of using intrinsic 4H-SiC substrate for CNT is to investigate the influence of SiC substrate on the generation of photocurrent in a physical device. Because generation of photocurrent is also influenced by the plasmon-phonon interaction of graphene and insulated substrates. Plasmon-phonon mode can be excited in graphene-based devices in the mid-infrared region under s-polarization. This interaction increases the overall temperature of interacting phonons and electrons and results in an increase in photo-generated current in graphene-based devices [52,53]. But as mentioned above, due to some limitation of simulator in controlling polarization of incident light, high values of photo-generated current could not be achieved in the presented work. In the near future, we want to investigate this plasmon-phonon coupling mode in case of SiC substrate in a physically fabricated device.

We next intend to create the physical fabrication of this simulated device and implement both detection mechanisms in that device. Our plan is to use a locally developed amplifier [54] to remove noise signal and extract the correct values of photocurrents from the CNT-based sensor. In this experiment, two strips of this same CNT-based devices will be placed parallel to each other. One device will be kept in dark condition while the other one will be illuminated with a light source. Both signals will be fed to the amplifier to detect the change in photocurrent and get rid of noise signal.

## 4. Conclusions

The purpose of the work presented in this article is to access the feasibility of CNT as a benzene and hydrogen-sulfide molecule detector and introduce a new gas detection mechanism. Carbon nanotube (CNT)-based molecule detectors could be promising candidates to replace conventional solid-state sensors due to their small size, better sensitivity, low operating temperature, and low cost. In this article, nanoscale simulations of CNT-based benzene and hydrogen-sulfide sensors have been done. Single-wall CNT has been deposited on intrinsic 4H-SiC substrate to form a sensor. The change in density of states (DOS), electric current, and photocurrent have been calculated for the simulated carbon nanotube-based sensor. A significant change in DOS, electric current, and photocurrent in the presence of benzene and hydrogen-sulfide molecules have been observed. A novel molecule detection mechanism (change in photocurrent) along with the state-of-the-art molecule detection mechanism (change in electric current) for CNT-based sensors have been proposed to improve the sensor selectivity and accuracy. The simulation results revealed that different target molecules affected the DOS, electric conductivity, and photoconductivity of the device uniquely. This change in electric current as well as photocurrent in the presence of different target molecules can be used simultaneously to detect their presence effectively. Although due to some limitation of the simulator in controlling the polarization of incident light and other related parameters, high values of photocurrent could not be achieved. Still, CNT has been recognized an exciting material for photo detection applications. We next intend to physically fabricate this simulated device and implement our proposed molecule detection mechanism in that device.
